# Lack of infectivity of HBV in feces from patients with chronic hepatitis B virus infection, and infection using chimeric mice

**DOI:** 10.1186/s13104-015-1337-z

**Published:** 2015-08-20

**Authors:** Haruki Komatsu, Ayano Inui, Takeyoshi Murano, Tomoyuki Tsunoda, Tsuyoshi Sogo, Tomoo Fujisawa

**Affiliations:** Department of Pediatrics, Toho University Sakura Medical Center, 564-1 Shimoshizu Sakura, Chiba, Japan; Department of Pediatric Hepatology and Gastroenterology, Eastern Yokohama Hospital, Kanagawa, Japan; Department of Research and Development, Toho University Sakura Medical Center, Chiba, Japan

**Keywords:** HBV, Chimeric mouse, Selective vaccination, Feces, Body fluid

## Abstract

**Background:**

Body fluids such as saliva and tears from patients with hepatitis B virus (HBV) infection are known as infectious agents. The infectivity of feces from patients with HBV infection has not been established. The aim of this study was to determine whether feces from HBV carriers can be a source of HBV infection.

**Methods:**

Thirty-three children and 17 adults (ages 0–49 years, median age 13 years) who were chronically infected with HBV were enrolled. The levels of HBV DNA in the feces from these patients were quantified by real-time PCR, and the levels of fecal HBsAg were measured. Isolated human hepatocytes from chimeric mice with humanized livers were co-cultured with serum, tears and feces from the HBV carriers. Four chimeric mice were inoculated intravenously with sterilized feces from HBV carriers.

**Results:**

HBV DNA was detected in the feces of 37 (74 %) of the 50 patients. The fecal HBV DNA levels ranged from 2.8 to 8.4 log copies/mL (mean ± SD  =  5.6 ± 1.2 log copies/mL). A significant correlation was observed in the levels of HBV DNA between serum and feces (r  =  0.54, p < 0.05). Of the 13 HBV carries, 7 (54 %) were positive for fecal HBsAg. The fecal HBsAg levels ranged from 0.06 to 1.0 IU/mL (median 0.28 IU/mL). Immunogold electron microscopy showed Dane particles in feces. HBV DNA was detected in the human hepatocytes co-cultured with serum and tears, but not in those co-cultured with feces. HBV DNA was not detected in the serum of the chimeric mice after oral or intravenous inoculation with sterilized fecal samples, which contained 5 log copies/mL of HBV DNA levels.

**Conclusions:**

Although the positive rate of fecal HBV DNA was high, the fecal HBsAg levels were extremely low. The chimeric mice were not infected with HBV after oral or intravenous inoculation with sterilized fecal samples. Therefore, feces from HBV carriers seem not to serve as an infectious vehicle for the transmission of HBV.

## Background

Hepatitis B virus (HBV) infection remains a global health problem. HBV is transmitted either perinatally or horizontally. Percutaneous and mucosal exposures to infectious blood or other body fluids are the routes of transmission [1]. Chronic HBV inflammation of the liver can increase the risk of cirrhosis and hepatocellular carcinoma [1]. Hepatitis B vaccination is the most effective measure to prevent HBV infection. As of 2012, 181 (93 %) of the 194 World Health Organization (WHO) Member States have introduced hepatitis B vaccine into their routine childhood vaccine programs [2]. However, Japan, the United Kingdom, Denmark, Norway, and Sweden have been implementing a selective vaccination strategy on the basis of cost-effectiveness.

Although at-risk strategies are needed to evaluate the individual risk of HBV transmission, the definitions of high-risk persons or groups vary from country to country [3]. Individuals who experience close family contact with a person who has an acute and chronic hepatitis B infection comprise one of the high-risk groups that should be vaccinated [1, 3]. However, the mechanism underlying the transmission of HBV by close contact is unclear. Body fluids such as saliva, sweat, tears, urine and feces might be sources of HBV through close contact [4–13].

Since the Australia hepatitis B surface antigen (HBsAg) was discovered in 1965 [14], serum, saliva, semen, and tears have been demonstrated to be infectious via the use of animal models [12, 15–18]. Regarding feces, the results of several investigations are conflicting [13, 19–33], and no experimental transmission of HBV using an animal model had been performed. In some studies, HBsAg was detectable in the feces of patients with an acute and chronic infection [19, 21, 23, 24]. In contrast, other studies failed to identify HBsAg in the feces [25, 30, 33]. It was hypothesized that intestinal mucosa containing an inhibitor of HBsAg and bile salts could damage HBV in the gastrointestinal tract [26, 27, 29, 32]. However, these investigations were conducted in the 1970s, and the infectivity of feces had not yet been determined. Because a universal hepatitis B vaccination was recently introduced in almost all countries, little attention has been paid to the infectivity of feces from persons with an HBV infection. However, for the countries adopting an at-risk strategy of vaccination, the evaluation of the infectivity of feces is indispensable to control HBV infection.

In the present study, to answer the question of whether feces from HBV carriers are an infectious agent, we quantified the levels of HBV DNA and HBsAg in feces from patients with chronic HBV infections. We also conducted an experimental transmission of HBV derived from feces, using chimeric mice with severe combined immunodeficiency and carrying a urokinase-type plasminogen activator transgene controlled by an albumin promoter (uPA/SCID), with transplanted human hepatocytes [34]. The chimeric mice were orally and intravenously inoculated with HBV from feces.

## Methods

### Patients and materials

This was a prospective cohort study in which chronic HBV carriers were recruited at our outpatient clinic from March 2011 to April 2012. Their condition of chronic hepatitis B infection was routinely evaluated by blood examination. All of the patients were asymptomatic. The patients who had gastrointestinal diseases or received antiviral drugs were excluded. Feces were collected in preparation tubes by the patients and taken to the hospital by the patients. All of the feces were negative for the norovirus, rotavirus, and adenovirus rapid detection kits (Quick Navi-Noro, Otsuka, Tokyo; BD Rota/Adeno, BD Diagnostics, Tokyo). Samples of the patients’ serum, tears, and saliva were also collected, at our outpatient clinic. Samples were stored at −80 °C until assay was performed.

### HBV DNA extraction and real-time PCR assay

HBV DNA in the feces was extracted using the QIAamp DNA Stool Mini kit (Qiagen, Hilden, Germany). In the density gradients, the QIAamp DNA Mini kit (Qiagen) was used for the extraction of fecal and serum HBV DNA from each fraction fluid. The extracted DNA was dissolved in 100 µL of elution buffer. An in-house TaqMan real-time assay was used for the quantification of HBV DNA from feces [35]. HBV DNA extracted from each fraction in density gradients was also quantified by the in-house TaqMan real-time assay. A polymerase chain reaction (PCR) assay was performed in an MX3000P QPCR System (Agilent Technologies, Tokyo), and the results were analyzed with MxPro software (version 3.0). The lower detection limit was <100 copies/mL.

All assays were carried out in triplicate with negative control samples. The PCR assay was standardized using HBV DNA samples of known concentrations measured by COBAS TaqMan HBV DNA test version 2.0 (Roche Diagnostics, Tokyo) with a lower detection limit of 2.1 log copies/mL and recombinant plasmid controls. Therefore, the conversion factor between HBV copies/mL and HBV IU/mL is considered to be 5.82 copies/IU. Genotyping of the HBV was determined by the PCR-Invader assay [36].

### Filter-sterilized supernatant of the mixture with feces

Approximately 1000–2000 mg of feces were put into a 50-mL tube and mixed with 20 mL of phosphate-buffered saline (PBS) by vortex for 3 min. The 50-mL tube containing the mixture was centrifuged at 3000 rpm for 10 min at room temperature, and then the supernatant was put in 2-mL sample tubes. These 2-mL tubes were centrifuged at 14,000 rpm for 10 min at 4 °C, and the supernatant was collected again. Finally, the supernatant was filter-sterilized with a 0.2-µm filter. The filter-sterilized supernatant was used for the quantification of HBsAg, the density gradient, the co-culture using human hepatocytes isolated from a chimeric mouse, and the inoculation of chimeric mice with humanized liver.

### Purification and analysis of HBV in the density gradients

Discontinuous iodixanol (Optiprep, Axis-Shield, Oslo, Norway) density gradients (6, 10, 20, 30, 40, and 50 %) was prepared with a solution containing 60 mM Tris–HCl (pH 7.4), 6 mM EDTA, and 0.25 M sucrose. The volume of each iodoxanol-sucrose solution was 450 µL, and the total volume of the density gradients was 2.7 mL in a thick-walled polycarbonate centrifuge tube (Beckman Coulter; Brea, CA). One hundred microliters of a serum sample or a filter-sterilized fecal sample was layered onto the discontinuous iodixanol density gradients. The gradients were ultracentrifuged at 1,00,000 rpm at 4 °C for 4 h (Optima TLX, Beckman Coulter) and harvested manually from the top, collecting 15 fractions of 150 µL each from each sample. Fifty microliters of PBS was added to each fraction, and a total of 200 µL was applied for DNA extraction.

### Conventional and indirect immunogold labeling electron microscopy

For conventional electron microscopy (EM), a drop of the purified fecal HBV by the density gradients was placed on a carbon-coated 200-mesh copper grid (EM Japan, Tokyo), and the excess fluid was blotted with filter paper. The grid was then negatively stained with 2 % uranyl acetate. To confirm the existence of HBV in feces, we performed indirect immunogold labeling EM as described [37, 38]. In brief, a drop of the purified fecal HBV was placed on a carbon-coated 200-mesh nickel grid (JEOL, Tokyo) and incubated for 15 min at room temperature. The grid was then washed with PBS and floated on a drop of 4 % BSA for 5 min, followed by another washing with PBS. Next, the grid was incubated on a drop of primary antibody solution (human anti-HBsAg immunoglobulin, Nihon Pharmaceutical, Tokyo, diluted 1:30 in PBS) for 60 min and secondary antibody solution (goat polyclonal secondary antibodies to human IgG, 5 nm Gold; Abcam, Tokyo: diluted 1:100 in PBS) for 60 min. After the incubation with the secondary antibody solution, the grid was floated on a drop of 2 % glutaraldehyde for 15 min. Then, the grid was negatively stained with 2 % phosphotungstic acid. Samples were examined with an electron microscope (JEM-1200EX: Japan Electron Optics Laboratory, Tokyo).

### Hepatitis B surface antigen assay

Serum and fecal HBs antigen was quantified using the Architect HBsAg QT assay (Abbott Laboratories, Chicago, IL, USA), which is a chemiluminescent microparticle immunoassay. It is internally calibrated using the World Health Organization reference standard for HBs, and measures HBsAg concentrations within the range of 0.05–250 IU/mL. Samples with HBsAg levels above or below this range require a lower or greater dilution in the manufacturer’s diluent to bring them into the calibration range. The lower limit of detection is 0.05 IU/mL.

### Culture of isolated human hepatocytes co-cultured with HBV

To investigate the infectivity of HBV, we purchased fresh primary human hepatocytes from chimeric mice with humanized liver, which were severe combined immunodeficiency, carrying a urokinase-type plasminogen activator transgene controlled by an albumin promoter (uPA/SCID) with transplanted human hepatocytes, from Phoenix Bio (Hiroshima, Japan) [39–42]. Isolated hepatocytes were inoculated at 1–2 × 10^5^ cell/cm^2^ in 35-mm dishes in a 24-well plate, which was coated with type I collagen. The human hepatocytes in each well were cultured in 500 µL of DMEM medium (Life Technologies, Tokyo) supplemented with 10 % FBS (Life Technologies), 100 U/mL penicillin (Life Technologies), 100 µg/mL streptomycin (Life Technologies), 20 mM HEPES, 44 mM HCO_3_, l-proline 15 µg/mL (Sigma, St Louis, MO, USA), insulin 0.25 µg/mL (Sigma), 50 nM dexamethasone (Sigma), EGF 5 ng/mL (Sigma), 0.1 mM Asc-2p, and 2 % DMSO (Sigma) at 37 °C in a 5 % CO_2_-incubator. At day 0, 50 µL of serum and filter-sterilized body fluids (tears, saliva, and feces) from patients with chronic HBV infections was added to wells in which human hepatocytes from chimeric mice were present, with 500 µL of medium.

The human hepatocytes were co-cultured with HBV from the filter-filtrated body fluids (tear No. 1–10 and feces No. 1–6) for 24 h. On day 1 and day 2, 500 µL of medium change was carried out. The medium was then changed every 5 days during the culture. At the end of the culture, the hepatocytes were collected from the plates and put into a tube. The tube was centrifuged at 15,000 rpm for 5 min. The supernatant was removed from the tube, and the pellet was used for the quantification of HBV DNA levels in human hepatocytes. HBV DNA was extracted from the supernatant and hepatocytes using a commercial kit (QIAamp DNA Mini kit: Qiagen) and quantified by a real-time PCR assay [35].

### Inoculation of chimeric mice and real-time PCR for mice sample

Four female chimeric mice with humanized liver were purchased from Phoenix Bio., Ltd. (Hiroshima, Japan). Human hepatocytes were imported from BD Bioscience (Woburn, MA, USA). The data of the four chimeric mice (No. 101, 102, 201, 202) were as follows: body weight, 22.3, 20.1, 19.9 and 20.9 g; serum human-albumin levels; 7.5, 6.6, 8.3, and 7.2 mg/mL. Of the four mice, two (Nos. 101 and 102) were inoculated orally with 100 µL of the filter-sterilized fecal sample every day for 28 days. The remaining mice (Nos. 201 and 202) were intravenously inoculated with 100 µL of the filter-sterilized fecal sample once. The filter-filtrated feces (No. 7: HBV DNA = 5.9 log copies/mL, HBsAg = 0.14 IU/mL, genotype C) was used for the oral and intravenous inoculations. After the inoculations, blood samples for the real-time PCR assay were taken from the chimeric mice every week. Fifty microliters of whole blood samples were taken from the mice every week after the inoculation, and sera were separated. HBV DNA was extracted from 20 µL of mouse serum. The HBV DNA of the mouse serum was quantitatively measured using a real-time PCR as described [12]. These chimeric mice were kept in a clean room and supplied with sterilized laboratory chow and water. Mice were anesthetized with isoflurane and sacrificed.

### Statistical analysis

Non-categorical variables were compared between groups by Mann–Whitney U test. For the correlations between log HBV DNA in serum and feces, we used Pearson’s correlation coefficient. All tests were two-sided, and *p* values of 0.05 or less were considered significant. All statistical analyses were performed with StatMate IV for Windows (Advanced Technology for Medicine and Science, Tokyo) and Microsoft Office Excel 2007.

### Ethics statement

All animal experiments were performed in accordance with both the Guidelines for Animal Experimentation of the Japanese Association for Laboratory Animal Science and the recommendations in the Guide for the Care and Use of Laboratory Animals of the National Institutes of Health and under the approval of the Ethics Review Committee for Animal Experimentation of Phoenix Bio (No. 0809). The study protocols were approved by the ethical committee of Eastern Yokohama Hospital (No. 2011017) and performed in accordance with the ethical guidelines of the 1975 Declaration of Helsinki. Written informed consent was obtained from all parents or legal guardians prior to sample collection.

## Results

### Patients and materials

Between March 2011 and April 2012, 33 children and 17 adults (25 males, 25 females, age range 0–49 years; mean age ± SD, 17.1 ± 13.4 years; median age, 13 years), who were chronically infected with HBV were enrolled in this study. Of these 50 patients with chronic hepatitis B infections, 37 were positive for HBeAg. The HBV DNA levels in their serum ranged from 2.3 to >9 log copies/mL (>9 log copies/mL in 24 patients, 6–9 log copies/mL in 13 patients, and >2.1 to <6 log copies/mL in 13 patients). Six patients and 44 patients were infected with genotype B and genotype C, respectively.

### Positive rate of HBV DNA from feces

**Fig. 1:**
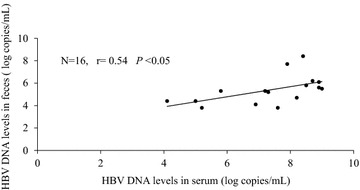
Data from patients whose levels of HBV DNA in serum ranged from 4.1 to 9.0 log copies/mL were used for the analysis. There was a significant correlation between HBV DNA in serum and saliva/tears (r = 0.54, p < 0.05)

HBV DNA was extracted from 50 to 220 mg of feces (solid sample) according the instruction manual of the commercial kit. HBV DNA was detected in feces in 37 (74 %) of the 50 patients by real-time PCR. The positive rates of fecal HBV DNA in the patient with serum HBV DNA >9.0 log copies/mL, 6.0–9.0 log copies/mL, and <6.0 log copies/mL were 86 % (21/24), 85 % (11/13), and 38 % (5/13), respectively (Table 1). The levels of HBV DNA levels in the feces ranged from 2.8 to 8.4 log copies/mL (mean ± SD  =  5.6 ± 1.2 log copies/mL). None of the patients in whom the levels of serum HBV DNA were less than 4.1 were positive for fecal HBV DNA. Because the upper detection limit of the COBAS TaqMan HBV DNA test was more than 9 log copies/mL, we used the data from the patients in whom the levels of HBV DNA in serum ranged from 4.1 to 9.0 log copies/mL. Data from 16 patients were available for the correlation analysis of HBV DNA levels between serum and feces [(HBV DNA levels in feces)  =  2.08  +  0.59  ×  (HBV DNA levels in serum)]. A significant correlation was observed in the levels of HBV DNA between serum and feces (r = 0.54, p < 0.05; Fig. 1).

**Table 1:** The positive rate of fecal HBV DNA

HBV DNA levels in serum (log copies/mL)	>2.1–<6	6.0–9.0	9.0<
Positive/total number (%)	5/13 (38)	11/13 (85)	21/24 (86)

### Quantification of fecal HBsAg

**Fig. 2:**
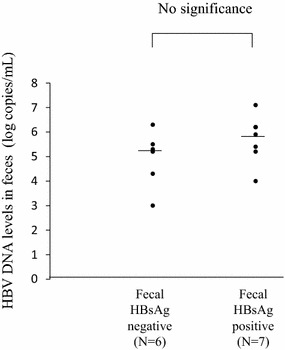
There was no significant difference in fecal HBV DNA levels between the HBs negative samples (serum HBV DNA levels: range from 3.0 to 6.3 log copies/mL, mean ± SD, 4.9 ± 1.1 log copies/mL; median, 5.3 log copies/mL) and HBsAg positive samples (HBV DNA levels: range from 4.0 to 7.1 log copies/mL, mean ± SD, 5.7 ± 1.0 log copies/mL; median, 5.9 log copies/mL)

Thirteen HBV DNA-positive fecal samples (serum HBV DNA levels: >9.0 log copies/mL, n = 9; 7.0–9.0 log copies/mL, n = 4) were available for the measurement of HBsAg levels. The levels of fecal HBV DNA ranged from 4.5 to 7.1 log copies/mL (mean ± SD; 5.4 ± 1.1 log copies/mL; median, 5.4 log copies/mL). Of the 13 feces samples, 7 (54 %) were positive for HBsAg. The levels of HBsAg ranged from 0.06 to 1.0 (mean ± SD, 0.35 ± 0.32 IU/mL; median, 0.28 IU/mL). There was no significant difference in fecal HBV DNA between the HBsAg-negative patients (n = 6, fecal HBV DNA levels: range from 3.0 to 6.3 log copies/mL, mean ± SD; 4.9 ± 1.1 log copies/mL; median, 5.3 log copies/mL) and the HBsAg-positive patients (n = 7, fecal HBV DNA levels: range from 4.0 to 7.1 log copies/mL; mean ± SD; 5.7 ± 1.0 log copies/mL; median, 5.9 log copies/mL) (Fig. 2). We tested 5 HBV DNA-negative fecal samples for HBsAg. All of them were negative for HBsAg.

**Fig. 3:**
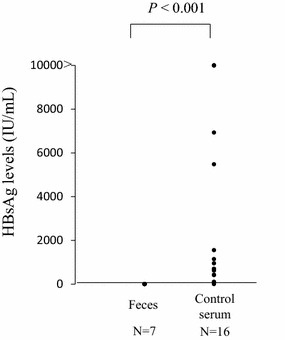
The levels of HBsAg in the serum were significantly higher than those in the feces (p < 0.001)

Because the levels of fecal HBsAg were low, we performed a comparison of HBsAg levels between serum and feces. Sixteen serum samples (serum HBV DNA levels: range from 4.3 to 6.0 log copies/mL; median, 5.0 log copies/mL, HBsAg levels: range from 2.6 to 10,000< IU/mL; 824 IU/mL) were prepared as controls for the comparison with the HBsAg levels in the fecal samples. The results of the comparison are shown in Fig. 3. Although there was no significant difference in HBV DNA levels between the control serum and feces, a significant difference was observed in the HBsAg levels between the serum and feces. These findings suggested that there is a discrepancy in levels between viral DNA and viral protein.

### Centrifugation and electron microscopy

**Fig. 4:**
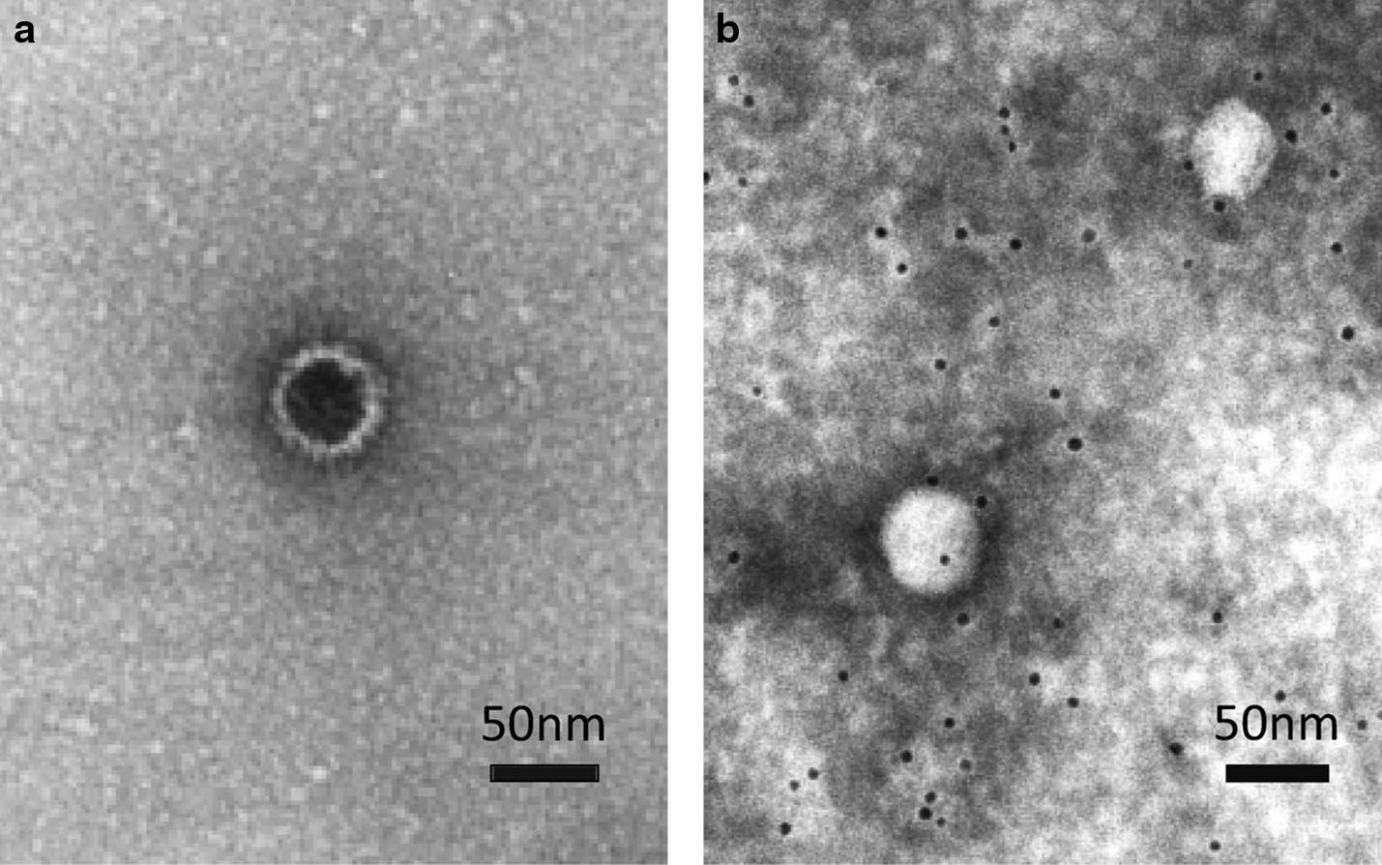
**a** Conventional electron micrograph of a Dane particle (stained with 2 % uranyl acetate). **b** Indirect immunogold labeling electron micrograph of Dane particles and spherical particles using human IgG antibody to HBsAg and goat polyclonal secondary antibody to human IgG-conjugated colloidal gold particles (5 nm) (stained with 2 % phosphotungstic acid)

To determine which fraction contains adequate levels of HBV DNA for EM, we used three serum samples (HBV DNA levels: 8.6, 9.0, and 5.8 log copies/mL) and three fecal samples (HBV DNA levels: 5.9, 6.2, and 5.3 log copies/mL) for the ultracentrifugation. After centrifugation, serum and fecal HBV DNA was extracted from each fraction, and HBV DNA was then quantified by real-time PCR. Both the serum and feces showed a peak of HBV DNA levels in fraction Nos. 7–8. We thus used the No. 8 fraction of Fecal No. 1 sample (fecal HBV DNA levels: 7.1 log copies/mL, HBsAg levels: 0.14 IU/mL) for the EM. A Dane particle (42 nm dia.) was observed by conventional EM (Fig. 4a). Dane particle (42 nm dia.) and spherical particles exhibiting specific gold labeling after reacting with human IgG antibody to HBsAg were observed by immunogold EM (Fig. 4b).

### Infectivity of HBV in the isolated human hepatocytes

We evaluated serum, tears, and feces for infectivity as an infectious vehicle of HBV. Initially, we co-cultured isolated human hepatocytes with two serum samples (HBV DNA levels: serum 1, 8.7 log copies/mL; serum 2, 8.6 log copies/mL) as positive controls (Table 2). HBV DNA was detectable in the culture supernatant in all of the wells until day 92 of inoculation. In addition, the HBV DNA levels in the human hepatocytes were higher than that in the culture supernatant at the end of culture (day 92). These findings suggested that HBV could replicate in the isolated human hepatocytes and that this culture system is a useful tool for infectious experiments.

**Table 2:** HBV DNA levels in isolated human hepatocytes co-culture with serum, tears, and stool

Sample (pre-inoculation)	Day 1	Day 2	Day 7	Day 12	Day 17	Day 22	Day 27	Day 72	Day 92	Cells
Serum 1 (8.7)	7.5	6.2	5.5	5.3	5	4.7	4.7	4.5	2.6	6.1^a^
Serum 2 (8.6)	7	6.6	6.5	6.8	5.4	5.3	5.5	5.7	5.3	6.8^a^
Tear 1 (5.3)	3.5	Neg	Neg	Neg	Neg	Neg	3.1	Neg	Neg	2.6^a^
Tear 2 (6.0)	4.4	3.9	4.5	4.2	4	2.8	3.1	Neg	Neg	3.2^a^
Tear 3 (5.6)	3.1	2.7	Neg	Neg	Neg	Neg	2.7	Neg	Neg	2.8^a^
Tear 4 (5.7)	3.7	3.2	Neg	2.9	Neg	Neg	Neg	Neg		Neg
Tear 5 (5.8)	4.9	4	2.4	2.2	Neg	Neg	Neg	Neg		3.6
Tear 6 (6.1)	5.2	4.6	3.7	2.8	3.6	2.8	2.2	Neg		NA
Tear 7 (5.7)	5.7	4.9	4.6	4.4	Neg	Neg	Neg	Neg		3.5
Tear 8 (6.9)	6	5.5	5.2	4.6	4.9	Neg	Neg	Neg		4.6
Tear 9 (5.6)	4.3	4.6	Neg	Neg	Neg	Neg	Neg	Neg		Neg
Tear 10 (5.9)	4.9	4.8	Neg	Neg	Neg	Neg	Neg	Neg		Neg
Feces 1 (7.1)	6.3	6.1	5.3	4.6	4.6	4.2	Neg	Neg		Neg
Feces 2 (6.3)	5.6	4.3	4.1	4.6	4.1	4.2	Neg	Neg		Neg
Feces 3 (6.5)	5.9	5.7	5.5	5.1	4.6	Neg	Neg	Neg		Neg
Feces 4 (6.8)	6.2	6.5	5.9	5.4	5.3	4.7	4.4	Neg		Neg
Feces 5 (6.9)	6.6	6.1	5.6	5	4.7	Neg	Neg	Neg		Neg
Feces 6 (6.4)	5.9	5.7	5.4	4.7	4.5	Neg	Neg	Neg		Neg

HBV DNA units: log copies/mL

^a^HBV DNA levels in hepatocytes was measured at 92 day of inoculation

With regard to tears, we co-cultured human hepatocytes with tear samples Nos. 1, 2, and 3 until day 92 and tear Nos. 4–10 until day 72 of inoculation (Table 2). Of the 10 tears, four (tears 1, 2, 3, and 6) continued to be positive for HBV DNA in the culture supernatant until day 27 of inoculation. Although all of the supernatants of hepatocytes co-cultured with tear samples became negative for HBV DNA at the end of the culture, HBV DNA was detected in the hepatocytes co-cultured with six tear samples (tear 1 at day 92, tear 2 at day 92, tear 3 at day 92, tear 5 at day 72, tear 7 at day 72, and tear 8 at day 72). In contrast to the serum and tears, HBV DNA was not detected in any of the hepatocytes co-cultured with feces samples. These findings suggested that HBV in serum and tears has the potential to enter into hepatocytes and replicate in the hepatocytes, whereas HBV in feces does not have this potential.

### Infectivity of HBV from feces in chimeric mice

The two mice (Nos. 101 and 102) were inoculated orally every day for 28 days. However, HBV DNA was not detected in their serum for 10 weeks after the inoculation (1st inoculation). Similarly, the remaining mice (Nos. 201 and 202), which inoculated intravenously once, did not become positive for serum HBV DNA for 10 weeks after the inoculation (1st inoculation). Because all four of the mice remained negative for 10 weeks after inoculation, we changed the initial plan of this experiment. We prepared two other filter-sterilized fecal samples (feces 8: HBV DNA = 5.3 log copies/mL, HBsAg <0.05 IU/mL, genotype C; feces 9: HBV DNA = 5.4 log copies/mL; HBsAg = 0.46 IU/mL, genotype C) for the 2nd inoculation. Because the mice (Nos. 201 and 202) had already died before the 2nd inoculation, Nos. 101 (feces 8) and 102 (feces 9) were inoculated intravenously with 100 µL of the filter-sterilized fecal sample once (2nd inoculation). One mouse died 10 weeks after the 2nd inoculation and the other (No. 102) died 4 weeks after the 2nd inoculation. Neither of them became positive for serum HBV DNA until they died. The results of serum HBV DNA in the mice are summarized in Table 3.

**Table 3:** Serum HBV DNA levels in chimeric mice after inoculation with feces

Mode of administration	Chimeric mice			
	No. 101	No. 102	No. 201	No. 202
Oral administration	Neg (1st inoculation)	Neg (1st inoculation)	ND	ND
Intravenous administration	Neg (2nd inoculation)	Neg (2nd inoculation)	Neg (1st inoculation)	Neg (1st inoculation)

*Neg* negative for HBV DNA, *ND* not done

## Discussion

For the past three decades, the infectivity of feces from patients with HBV infection has been a controversial topic. In the present study, to clarify the infectivity of HBV in feces, we used advanced technologies such as real-time PCR, immunogold EM [37, 38], the quantification of HBsAg [43, 44], fresh primary human hepatocytes from chimeric mice [40–42], and chimeric mice with humanized liver [34]. Although HBV DNA was detected by conventional PCR in chimpanzees [45], to the best of our knowledge, the present study is the first time that HBV DNA levels and HBsAg were quantified in feces from humans with HBV infections. The positive rate of HBV DNA in feces from the patients whose levels of serum HBV DNA were more than 6 log copies/mL was 86 % (32/37). This positive rate is comparable to those of saliva and urine, which were reported in our previous study [12]. Moreover, the mean levels of fecal HBV DNA were the same as that of saliva [12]. Because saliva has been demonstrated to be an infectious agent of HBV infection [15, 18], these findings suggest that feces contain sufficient amounts of HBV DNA to infect humans.

However, the results of the quantification of HBsAg threw some doubt on the infectivity of feces from HBV carriers. Approximately one-half of HBV DNA-positive feces were negative for HBsAg. The positivity of HBsAg was not associated with the levels of fecal HBV DNA. Additionally, the levels of HBsAg were extremely low in all of the HBsAg-positive feces. The feces from HBV carriers showed a discrepancy between HBV DNA levels and HBsAg levels. These findings can explain why the results of the studies conducted in the 1970s were conflicting [13, 19–23, 25–28, 30–32]. Low levels of HBsAg might cause conflicting data. High HBV DNA levels and low HBsAg levels might support the hypothesis that enzymes from intestinal mucosa and bacteria in the gastrointestinal tract could inactivate or damage HBV virions [27–29, 32]. HBV DNA might be relatively resistant to enzymes and bacteria and preserved in the gastrointestinal tract. In contrast, the envelope protein of HBV could easily incur damage in this environment, and the antigenicity of HBV is lost. The HBV envelope protein has a central role to play in the initial phase of hepatocyte infection, such as in the attachment to the cell surface, uncoating, and entry [46–48]. The change in the antigenicity of HBV thus results in the decrease of infectivity. However, we do not have any evidence suggesting this speculation.

If the HBV virion is damaged in the gastrointestinal tract, morphological changes of HBV could be observed. To clarify whether morphological changes of HBV are present, we used EM. Dane particles were detected in the sample isolated from feces by conventional EM. However, no obvious morphological change was observed by conventional EM. To confirm the presence of Dane-like particles in the feces, immunogold labeling EM was also performed. Dane particles exhibiting specific gold labeling were detected by EM. The shape of the Dane particles showed a slight morphological change. However, it was difficult to determine whether the change was due to the artifact of immunogold EM, and thus we could not draw a conclusion regarding whether HBV virions show morphological changes in feces. Beside infectious virions, HBV produces at least two other types of particles, subviral empty particle and subviral naked nucleocapsids particles [49]. It is an attractive hypothesis that fecal HBV DNA detected is related to naked nucleocapsids but not virions. It remained uncertain whether the conditions of this density gradient allow the separation of enveloped Dane particles from DNA-containing naked nucleocapsids. However, another method such as high-resolution CsCl density gradient was not performed to distinguish between nucleocapcids and virion in this study. Although only two particles were detected in Fig. 4b, goat secondary antibody to human IgG-conjugated colloidal gold was attached to the surface of two particles. We thought that these are Dane particles. However, further studies are necessary to confirm that fecal HBV DNA is related to naked nucleocapsids or virion.

Human hepatocytes isolated from chimeric mice with humanized livers recently became available for in vitro study [40–42]. Ishida et al. [42] confirmed the expression of HBV viral protein in human hepatocytes and the presence of extracelluar HBV DNA and HBsAg in vitro using human hepatocytes from chimeric mice using. Enzyme induction studies as well as hepatitis virus infection studies can be performed in vitro using this system. In the present study, the infectivity of serum, tears, and feces was evaluated using human hepatocytes isolated from chimeric mice. We found that although the HBV DNA levels in the supernatant were gradually decreased in the hepatocytes co-cultured with serum, the HBV DNA had been detectable for 3 months in the supernatant. Moreover, the HBV DNA levels in the hepatocytes were higher than those of the supernatant at the end of the culture period.

Although chimeric mice are much better than isolated hepatocytes regarding the efficiency of the viral replication [50], the present findings indicate that human hepatocytes isolated from chimeric mice were useful for the evaluation of viral infectivity. Unfortunately, the levels of pre-inoculation HBV DNA in the tears and feces were lower than that of the serum. Therefore, the supernatant of the hepatocytes co-cultured with tears and feces became negative for HBV DNA at 2 months after inoculation. However, HBV DNA was detected in isolated human hepatocytes co-cultured with 6 of 10 tears samples at the end of the culture period. In contrast to serum and tears, HBV DNA was not detected in any isolated human hepatocytes co-cultured with feces. These findings suggest that both serum and tears contain HBV virions which can infect human hepatocytes, but feces do not have them.

Covalently closed circular DNA (cccDNA) is indispensable for viral replication [46, 47]. Although the original in vitro study of HBV using human hepatocytes from chimeric mice detected cccDNA in hepatocytes [40], the present investigation failed to detect cccDNA in hepatocytes by real-time PCR. Moreover, the original study confirmed that HBV core proteins were specifically expressed in the human hepatocytes by immunostaining and lamivudine treatment reduced the HBV DNA levels in the culture medium and in the isolated human hepatocytes [40].

We used chimeric mice with humanized livers for the evaluation of the infectivity of feces. Chimeric mice have the most powerful ability to replicate HBV. Intravenous administration with at least 10–100 copies of HBV DNA has been demonstrated to infect chimeric mice [50]. The inoculated mice become positive for serum HBV DNA 4–5 weeks after inoculation [50]. In the present study, a total of four mice were administered 100 µL of sterilized feces intravenously. A total of three fecal samples were used for inoculation. The levels of HBV DNA in the three samples were more than 5 log copies/mL (equivalent to more than 10^4^ copies), which were sufficient levels of HBV DNA to infect chimeric mice. Two of the three samples were positive for HBsAg. However, three mice did not become positive for serum HBV DNA within 10 weeks after the intravenous inoculation, and the remaining mouse did not become positive within 4 weeks after the intravenous inoculation. These findings strongly indicate that feces from HBV infection are not infectious materials.

There are several limitations in the present study. This study is lack of sufficient data about sensitivity of cell-culture system. Only two serum samples were used as positive controls in this co-culture system. Ishida et al. reported that the threshold of the co-culture system was 10^5^–10^6^ copies/mL of serum HBV DNA. Thus, we used the patients’ serum containing 10^8^ copies/mL of HBV DNA as positive controls. Moreover, HBV infection events on single cell were not detected by immunostaining in this study. Immunostaing with more adequate positive controls and negative controls (e.g. inhibition experiments with antibodies or entry inhibitors) are needed to confirm the infectivity of fecal HBV in this cell-culture system. Compare to serum samples, the viral load was low in the fecal samples. We tried to concentrate the viral titers using protein concentration columns, but failed to concentrate them. Polyethylene glycol is helpful for concentrating and isolating viruses. However, we did not use it. The filter-sterilized supernatant of feces was used for the quantification of fecal HBsAg. But the recovery rate of HBsAg from feces was not evaluated. At least 10 chimeric mice including positive controls are required to confirm the infectivity of feces from HBV carrier. In addition, there was a plan whether infectivity of patient’s serum incubated with healthy control’s feces would be lost. However, financial condition did not allow us to perform these experiments. HBsAg and HBeAg secreted from cells were reliable markers to indicate the infection of HBV. Because the levels of HBV DNA were very low in the medium of isolated liver cells, we thought that HBsAg levels were under the detection limit. Thus, we did not measure the levels of HBsAg and HBeAg.

## Conclusions

In conclusion, the positive rate of fecal HBV DNA was similar to those of other body fluids in the patients with chronic HBV infection. However, we observed a discrepancy between HBV DNA levels and HBsAg levels in the patients’ feces. Chimeric mice with humanized livers were not infected with HBV by the intravenous administration of sterilized feces. Feces from patients with HBV infection seem not to be infectious agents.

## Abbreviations

HBeAg: hepatitis B e antigen
HBsAg: hepatitis B surface antigen
HBV: hepatitis B virus
PCR: polymerase chain reaction
